# Efficient Antifouling Surface for Quantitative Surface Plasmon Resonance Based Biosensor Analysis

**DOI:** 10.1371/journal.pone.0044287

**Published:** 2012-09-12

**Authors:** Claude Nogues, Hervé Leh, Joseph Lautru, Olivier Delelis, Malcolm Buckle

**Affiliations:** 1 LBPA, ENS de Cachan, CNRS, Cachan, France; 2 Institut d’Alembert, ENS de Cachan, Cachan, France; Semmelweis University, Hungary

## Abstract

Non-specific binding to biosensor surfaces is a major obstacle to quantitative analysis of selective retention of analytes at immobilized target molecules. Although a range of chemical antifouling monolayers has been developed to address this problem, many macromolecular interactions still remain refractive to analysis due to the prevalent high degree of non-specific binding. In this manuscript we explore the dynamic process of the formation of self-assembled monolayers and optimize physical and chemical properties thus reducing considerably non-specific binding while maintaining the integrity of the immobilized biomolecules. As a result, analysis of specific binding of analytes to immobilized target molecules is significantly facilitated.

## Introduction

While tremendous progress in developing antifouling surfaces has been achieved, the control of the integrity of biomolecules immobilized on surfaces has to be questioned. In particular, the dynamics of protein/DNA interactions is often studied by immobilizing known sequences of short DNA (up to 100 base pairs) on a surface and introducing, via a fluidic system, a diluted protein to the biochip surface. A frequently used method for immobilizing double stranded DNA (dsDNA) on a biochip surface consists of first adsorbing a single stranded DNA (ssDNA), modified with an active group at the 5′ or 3′ end, allowing the formation of a monolayer on a given substrate [1]. Subsequently, *in situ* hybridization is carried out with the complementary strand. Under these conditions, the maximum relative amount of resulting dsDNA rarely exceeds 50% [2]. However the alternative option of directly immobilizing short dsDNA directly on surfaces often leads to partial or complete denaturation [3], [4]. As a result, short DNA monolayers are composed of a mixture of dsDNA and ssDNA. The relative amount of dsDNA and ssDNA depends strongly on the DNA length, sequence and density once adsorbed on the surface. The buffer composition and the pH used for adsorption are also important parameters [1], [2]. As a consequence, proteins that are presented to the biochip surface will interact with both dsDNA and ssDNA. The measured apparent kinetic rate constant of a dsDNA binding protein interacting with a nucleic acid such as DNA varies significantly depending on the hybridization state of the DNA. Thus, kinetic measurements made on a mixed population of ssDNA and dsDNA would contain binding constants to both forms of DNA as well as any non-specific binding to the surface itself. Most analyses of kinetic data assume a Langmuir type adsorption-binding model [5]. Although non-specific interactions at the target molecules can be corrected by the use of multiple binding models, this often introduces errors in assigning numerical values to apparent binding constants. As a result, this precludes accurate quantitative analysis that would provide useful kinetic or affinity data. This last point is extremely important because additional difficulties arise from proteins that often form very stable complexes with non-specific sequences along the adsorbed dsDNA.

Surface Plasmon Resonance (SPR), one of the most established label-free biosensor techniques, measures changes in refractive index and thus changes in mass as a molecule is trapped at a surface generally through a mechanism involving a target or bait molecule immobilized at the surface [6]. Surface Plasmon Resonance imagery (SPRi), a more recently developed approach, allows analysis of the entire surface upon which discrete spots of ligands are immobilized [7]. However, by its very nature, the ensuing measurement, using the change in refractive index, cannot distinguish between molecules that are retained either specifically or non-specifically at the target molecule or non-specifically adsorbed onto the surface surrounding target molecules.

For non-specific interactions of proteins directly with the surface, the general strategies adopted to thwart this limitation carry out direct subtraction from target surfaces of signals from non target-containing surfaces. The kinetics of adsorption and desorption of non-specifically bound proteins to the surface differ significantly from specific interactions of proteins with immobilized target. Therefore, a simple subtraction of the reference potentially may modify the shape of kinetic curves, thereby introducing errors in the determination of binding constants [8]. A common surface used in SPR and notably the Biacore configuration involves a 100 nm thick dextran based polymer generally with carboxyl groups for convenient functionalization. Although this layer of dextran allows a high density of binding it was not chosen for this study for the following reasons, a) molecules that are bound are anisotropic and their density and orientation cannot be accurately controlled, b) the negative charge on the carboxyl groups can be involved in high non-specific ionic interactions with target molecules, thus being particularly fastidious with DNA binding proteins that often use initially an electrostatic interaction during the binding process, c) immobilised molecules are non homogenously distributed throughout a layer that extends 100 nm into the evanescent wave thus the resonant response represents an averaging over a thickness almost equivalent to a third of the penetration depth of the evanescent wave. Since all of these three points represent major stumbling blocks to the analysis of the formation of nucleoprotein complexes we decided to develop an appropriate and reliable surface to obviate these restrictions.

To date, the most common technique used to reduce non specific adsorption of proteins to a surface involves a coating of poly(ethylene glycol) (PEG) [9]. An important development of protein-resistant materials came from the work of Prime and Whitesides [10] who demonstrated that self-assembled monolayers (SAMs) of ethylene glycol oligomer terminated alkanethiols (OEG-SH) confer protein resistance to gold, as determined by SPR. OEG-SH SAMs on gold exhibit significantly improved resistance to non-specific protein binding compared to grafted PEG and provided important insights concerning the anti-fouling properties of PEGylated surfaces in general [10], [11], namely that long-chain PEGs are not required to limit protein adsorption and that the antifouling properties of OEG-SH SAMs on gold are controlled by the terminal hydrophilicity of the head group combined with the formation of a dense but disordered OEG brush with significant penetration of water into the OEG-SH SAMs [12]. The antifouling properties of EG*4* SAMs have been observed only for monolayers containing defects allowing conformational freedom to the EG*n* present in grafted PEG coatings [13], [14].

In this work we describe a method allowing both the optimization of dsDNA monolayer formation and dsDNA accessibility to proteins while simultaneously preventing non-specific interactions of the protein with the entire surface. This original approach consists of pre-treating SPRi biochip surfaces [15] prior to dsDNA adsorption with a sparse self assembled monolayer composed of *n* ethylene glycol (*n* = 4, EG*4*) [13], [16]. As a result, the dsDNA accessibility for ligand binding and related reactions is increased and no denaturation of the dsDNA oligomer was observed upon adsorption [17]. Using this method we characterized nucleoprotein complex formation between Primate Foamy Virus Integrase (PFV-1 IN) and random sequence dsDNA using SPRi.

Although PFV-1 IN was shown to be soluble in diluted solution; it forms a higher order multimer or aggregate upon interacting with DNA. PFV-1 IN polymerization is mainly mediated by non-specific protein-protein interactions and occurs at internal DNA positions [18]. Despite the fact that the presence of these higher order complexes complicates the study of specific binding and thus the protein’s activity, results obtained by anisotropy based assays indicate that PFV-1 IN has a higher apparent affinity for cognate sequences. It should be noticed that these apparent affinities were measured at equilibrium with a high IN/DNA ratio (the lowest concentration was 100 nM of enzyme for 12 nM of DNA) and resulted in an apparent affinity constant between 0.1 and 1.5 µM according to the buffer salt concentration and for a DNA of 45 bp [18].

By combining the antifouling properties of EG*4* SAM and the dynamics of SAM formation (studied in detail using quantitative radioactive measurements and SPRi), we were able to measure extremely low apparent equilibrium dissociation constants for nucleoprotein complex formation between PFV-1 IN and a DNA target leading to a deeper insight into IN/DNA complex formation.

## Results

### Binding of PFV-1 in Protein to DNA Target Using SPRi

**Figure 1:**
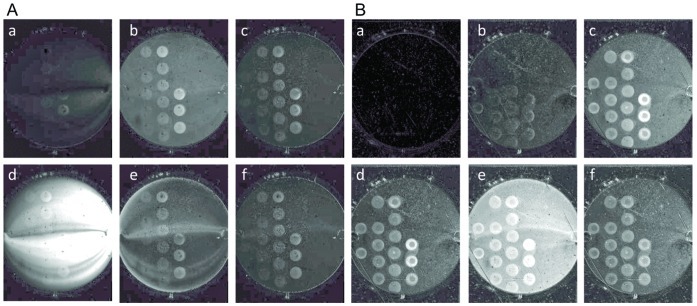
SPRi difference images of PFV-1 IN interacting with the biochip surface. ssDNA solutions at 50, 25, 10, 6, 3, 1, 0.5 and 0.1 µM were spotted in replicates on the pre-treated surface. The entire spot image was between 0.8 and 0.9 cm in diameter and each individual spot was between 400 and 450 µm in diameter. The spotting, of decreasing ssDNA concentration, starts from left to right, and downward. **A**: ssDNA spotted on EG4 pre-treated surfaces, PFV-1 IN at 200 nM was flowed across the SPRi surfaces containing the spotted ssDNA molecules. Difference images during the injection are shown in panels **a** and **b**, while panel **c** is a difference image after the injection of the protein was stopped. Panel **d** and **e** shows difference images during the injection of a solution of 0.1% SDS across the surface during the dissociation phase of PFV-1 IN. Panel **f** shows difference images of PFV-1 IN retained at the surface after the SDS injection. **B**: Surfaces were prepared as previously by spotting ssDNA at various concentrations on the pre-treated surface. Then the complementary ssDNA oligomers was flowed across the surface in order to hybridize and form dsDNA before injecting PFV-1 IN at 200 nM across the SPRi surface. Panels **a** to **c** are difference images of PFV-1 IN flowing across the SPRi surface and panel d is a difference image after the injection of the protein was stopped. Panel **e** and **f** are difference images during and after the injection of a 0.1% SDS solution.

Two strategies are commonly used to prepare dsDNA target monolayers on gold surfaces: immobilization of thiolated ssDNA (SH-ssDNA) and subsequent *in situ* hybridization or direct spotting of thiolated dsDNA (SH-dsDNA) on the surface. The former offers the advantage of allowing direct calculation by SPRi of the amount of dsDNA available as targets for subsequent interactions but suffers from the high probability that a number of SH-ssDNA molecules will remain non-hybridized on the surface as discussed in the introduction and in [2]. SPRi will thus be unable to distinguish PFV-1 IN binding to ssDNA as opposed to binding to dsDNA [19], [20]. This last point was demonstrated when SH-ssDNA was immobilized onto the EG*4* pre-treated gold surface (see experimental methods) and PFV-1 IN (200 nM) was flowed across the surface (Figure 1A, panels a and b). The change of the background colour (from black to grey) is a combination of the bulk change of the refractive index due to the presence of glycerol (10% v/v) and 1 M NaCl in the stock protein solution and non-specific interactions. The increase of the contrast between the ssDNA spots and the surface throughout the association phase (Figure 1A, panels a and b), confirms the efficiency of the EG*4* treatment, even at low density to prevent non-specific binding of the protein to the surface. In addition, this clearly demonstrates a significant interaction between PFV-1 IN and ssDNA. The increased signals seen as a function of spotted SH-ssDNA concentration, and thus SH-ssDNA density on the surface strengthens this last point (in Figure 1 the surface contains four rows of DNA spots with increasing DNA concentration going from left to right and downward). Furthermore, the PFV-1 IN/DNA complex is stable as a clear signal is still detected after the surface was rinsed with the running buffer for a few minutes after the protein injection was stopped (Figure 1A, panel c). Surprisingly, regeneration of the surface, i.e. removal of PFV-1 IN bound to the ssDNA, was not possible with 0.1% SDS, as residual PFV-1 IN molecules were retained on the ssDNA containing spots (Figure 1A, panels d to f). In view of these results we recreated a ssDNA-containing surface across which were flowed complementary ssDNA oligomers to hybridize and form dsDNA on the surface. As seen in Figure 1B panels a to c, PFV-1 IN bound specifically to target DNA with an obvious dependence on the DNA monolayer density. In addition, the intensity detected at each DNA spots is significantly stronger for PFV-1 IN interacting with dsDNA (Figure 1B, panels a to d) than for PFV-1 IN interacting with ssDNA (Figure 1A, panels a to c). Again, however, regeneration with 0.1% SDS failed to remove a large part of the PFV-1 IN retained at the DNA spots (Figure 1B, panels e and f). Since PFV-1 IN forms extremely stable complexes with ssDNA and *in situ* hybridization is incomplete, we investigated conditions to create homogenous dsDNA surfaces allowing accurate characterization of PFV-1 IN/DNA interactions using SPRi.

#### Self-Assembled monolayer of dsDNA on gold surface

Direct immobilization of short dsDNA on gold surfaces was shown to lead to its partial or complete denaturation [3], [4]. We attempted to prevent dsDNA denaturation by protecting the gold surface with a sparse self-assembled monolayer composed of EG*4* [13], [16] prior to overnight dsDNA adsorption.

**Figure 2:**
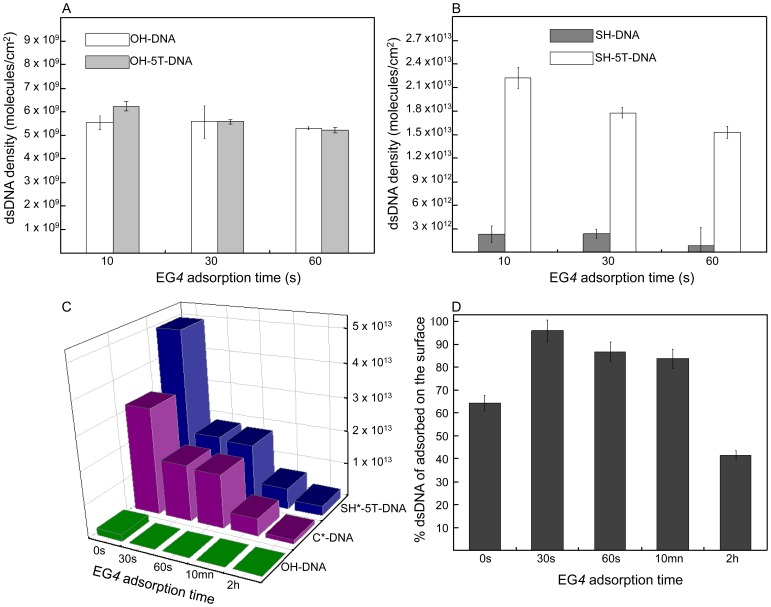
Densities of DNA immobilized on pre-treated surfaces. The amount of DNA retained at the surfaces was calculated as described in the experimental section. **A**: Density of dsDNA with (OH-5T-dsDNA) and without (OH-dsDNA) the T_(5)_ spacer on pre-treated surface. **B**: Density of the thiolated dsDNA with (SH-5T-dsDNA) and without (SH-dsDNA) the T_(5)_ spacer on pre-treated surface. **C**: Density of immobilized DNA on the pre-treated gold surface as a function of the EG4 SAM immersion time ranging from 30 s to 2 hrs. SAM adsorption time = 0 corresponds to the bare gold surface. The SH*-5T-dsDNA and C*-dsDNA indicates which of the DNA strands were ^32^P radiolabelled, namely the thiolated and the complementary strand respectively. OH-dsDNA refers to a ^32^P radiolabelled non-thiolated dsDNA. All the dsDNA strands have the same sequence. **D**: Relative amount of double stranded DNA adsorbed on the surface after the pre-treated gold surface was immersed in 10 µM dsDNA solutions. The % dsDNA adsorbed is calculated from the ratio between C*-dsDNA and SH*-5T-dsDNA, 100% corresponding to the SH*-5T-dsDNA density. The error bars correspond to the dispersion after accumulating 8 experiments for each EG4 adsorption time.

Initially, we asked to what extent does the incubation time of the gold surface in EG*4* solution influence the density of DNA immobilization on the surface? Since the addition of a spacer between the thiol group and the dsDNA should promote the efficiency of DNA chemisorption through the EG*4* monolayer, we simultaneously studied the influence of a penta-thymine spacer (T_(5)_) (introduced between the thiol group and the 5′ end of the dsDNA) on the DNA density. Thymine was selected because it facilitates extension of the DNA away from the gold surface resulting in a brush-like conformation [21], [22]. In addition, the length of the spacer confers the required flexibility such that the thiol group can interact with the gold surface across the EG*4* monolayer without protruding from it. This last point is crucial to prevent PFV-1 IN binding non-specifically and irreversibly to ssDNA. Gold surfaces were incubated with EG*4* for time periods of 10 s, 30 s and 1 min before overnight incubation with ^32^P dsDNA labelled at the 3′ end of the thiolated strand with (SH-*5T*-dsDNA) and without (SH-dsDNA) the T_(5)_ spacer. To quantify non-specific interactions of dsDNA with the EG*4* pre-treated gold surface we incubated the non-thiolated dsDNA with (OH-*5T*-dsDNA) and without (OH-dsDNA) the T_(5)_ spacer. The amount of non-specific adsorption (Figure 2A) can be compared with the density of SH-*5T*-dsDNA and SH-dsDNA immobilized on pre-treated surfaces at each of the EG*4* time treatments (Figure 2B). While non-specific adsorption remains extremely low (with no influence of the T_(5)_ spacer), the density of both dsDNA’s (with and without the T_(5)_ spacer) adsorbed on the surface decreases as the EG*4* adsorption time increases (Figure 2B). In addition, the T_(5)_ spacer allows the formation of a significantly denser dsDNA monolayer (at 30 s of EG*4* incubation time: 1.85×10^13^molecules/cm^2^ and 3.37×10^12^molecules/cm^2^ with and without the T_(5)_ spacer respectively). As a result, for 30 s of EG*4* incubation time, non-specific adsorption represents approximately 0.17% of the total corresponding SH-dsDNA but only 0.03% of the total SH-*5T*-dsDNA (OH-dsDNA or OH-*5T*-dsDNA density is 5.56×10^9^molecules/cm^2^). Consequently since the density of the DNA immobilised is increased in the presence of a spacer even though the amount of non-specific binding remained constant then the percentage of non-specifically bound DNA clearly is expected to decrease, as was indeed the case.

It is important to note that on bare gold surfaces (time point 0 in Figure 2C), under our experimental conditions, 4% of the adsorbed dsDNA was not specifically adsorbed to the surface (SH-*5T*-dsDNA density on bare gold was approximately 5.0•10^13^molecules/cm^2^ compared with 2.0•10^12^molecules/cm^2^ corresponding to the retained amount of OH-dsDNA). The SH-dsDNA density characterized on bare gold is in good agreement with densities reported in similar studies [23], [24]. Therefore, both pre-treatment of gold surfaces with sparse EG*4* SAM and the presence of the T_(5)_ spacer greatly improve the specificity of binding of thiolated DNA to gold.

To quantify the extent of dsDNA denaturation upon adsorption on bare gold and on EG*4* pre-treated surfaces, the two single strands that compose the SH-*5T*-dsDNA were radiolabelled separately and independently. The density of the two dsDNA SAMs should be identical were denaturation to occur. Figure 2C reports on the density of dsDNA attached to the surface at each of the EG*4* time treatments when alternatively either the thiolated DNA strand (SH*-*5T-*dsDNA) or the complementary strand (C*-dsDNA) are labelled. On bare gold and on EG*4* pre-treated surfaces, the density of immobilized dsDNA is systematically higher when SH*-*5T-*dsDNA is adsorbed compared with C*-dsDNA. The difference between SH*-*5T-*dsDNA and C*-dsDNA density is greater on bare gold than on EG*4* pre-treated surfaces confirming the denaturation of dsDNA upon adsorption on gold surface and the protection conferred merely by the EG*4* surface pre-treatment. The relative amount of dsDNA on gold and pre-treated gold (corresponding to the ratio between C*-DNA and SH*-*5T*-DNA, 100% is equivalent to the SH-*5T*-DNA* density) is shown in Figure 2D. On gold, almost 40% of the total dsDNA denatured upon adsorption (EG*4* adsorption time = 0). 30 s of EG*4* adsorption time reduced denaturation down to 5%. In addition, reports have indicated that 85% of a 3′ end thiolated dsDNA (of comparable length) denature upon adsorption on bare gold surfaces [17], confirming the significant contribution of the T_(5)_ spacer to the stabilization of short dsDNA following immobilization. A reasonable explanation of this is that the spacer increases the distance of the DNA from the gold surface as well as increasing the density and thus enhancing the brush like structure of the monolayer, the sum effect therefore would be to obviate the denaturing effect of the gold surface. While the EG*4* adsorption time strongly affected the density of dsDNA, it is interesting to note that the amount of ssDNA retained (corresponding to the difference between SH-*5T*-dsDNA^*^ and C-dsDNA^*^) is independent of the EG*4* adsorption time and was approximately 1.5 10^12^molecules/cm^2^ (figure 2C). Accordingly, denaturation of dsDNA on the surface reached 60% for 2 h of EG*4* SAM adsorption time since the adsorbed SH-*5T*-dsDNA density is the lowest.

The EG*4* protocol involving 30 s of EG*4* incubation time and overnight incubation with SH-*5T*-dsDNA was adopted as the standard protocol for preparing biochip surfaces because non-specific adsorption of dsDNA is negligible and the dsDNA is most stable.

**Figure 3:**
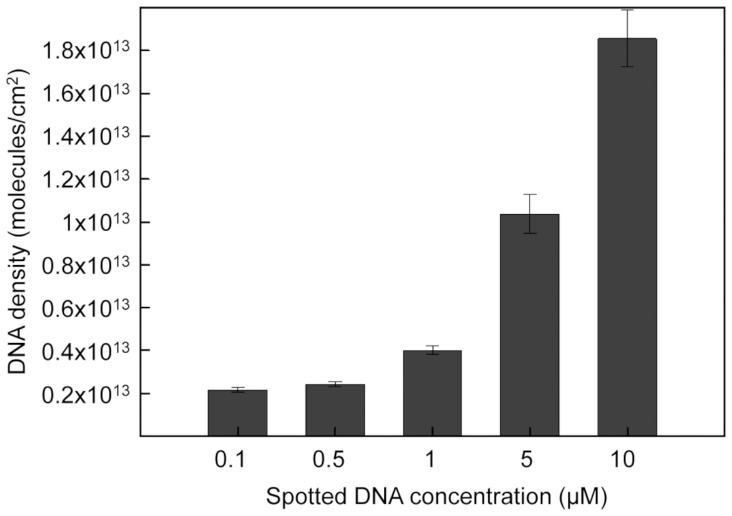
Density of DNA adsorbed on a pretreatedEG4 SAM as a function of the applied SH-5T-dsDNA concentration. The incubation time of the EG4 SAM is 30 s. The error bars correspond to the dispersion of the results over 4 separate measurements.

Judicious choice of the dsDNA concentration provides an additional parameter to control and optimize dsDNA density on the surface and thus improves dsDNA accessibility to proteins. Figure 3 shows that no dramatic increase in density is observed between 0.1 µM and 1 µM of SH-*5T*-dsDNA concentration applied. The dsDNA surface density also remained constant for applied SH-*5T*-dsDNA concentrations between 5 and 10 µM. However, a more dramatic variation was observed between applied SH-*5T*-dsDNA concentrations of 1 and 5 µM with approximately 4•10^12^molecules/cm^2^ at 1 µM and 1•10^13^molecules/cm^2^ at 5 µM (Figure 3).

#### Kinetics of PFV-1 IN/dsDNA complex formation and dissociation

**Figure 4:**
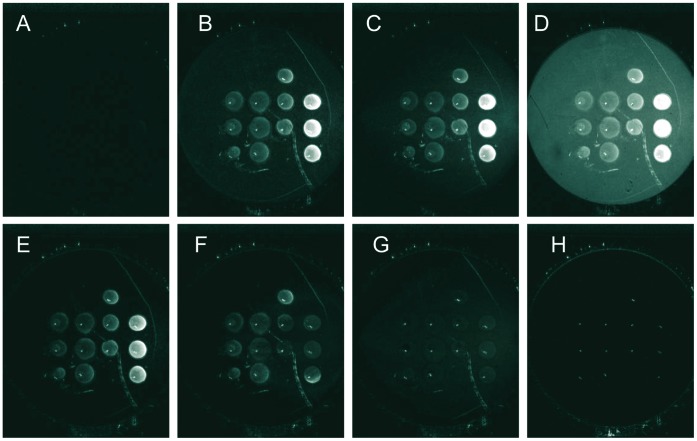
SPRi difference images of the biochip surface at different times during the course of the experiment. The EG4 adsorption time was 30 s. 4 different SH-5T-dsDNA solution concentrations were spotted in rows from right to left: 10, 5, 2.5 and 1 µM respectively. Each SH-5T-dsDNA concentration was spotted 3 times in the same column. **A**: t = 0 s, no PFV-1 IN protein injected. **B**, **C**, and **D** are images taken at t = 18 s, t = 3 min and t = 5 min respectively after 200 nM PFV-1 IN injection. **E** is the image taken 12 min after the injection was stopped. **F**, **G** and **H** are images taken at t = 9 s, t = 2 min, t = 6 min and t = 10 min after the start of the 0.1% SDS injection.

The differential images at different time points during injection of PFV-1 IN across the EG*4* pre-treated surface containing spots of immobilized target dsDNA are presented in figure 4. Different dsDNA concentrations (10, 5, 2.5 and 1 µM) were spotted in triplicate. Figure 4A corresponds to the differential image at time t = 0 where no protein was injected. Figures 4B to 4D are images taken during the association step, at a total PFV-1 IN protein concentration of 200 nM. Immediately following injection of the protein, the surface was continuously rinsed with buffer (Figure 4E). The background returned to its original value as opposed to the relative reflectivity intensity in the target dsDNA containing spots that remained high. Therefore, the non-specific interaction of the PFV-1 IN with the surface is not stable compared to the specific interaction of the PFV-1 IN with the dsDNA. It is important to note that the antifouling EG*4* SAM is identical both within and around the dsDNA spots thus ensuring that even within the dsDNA spots very little non-specific interaction with the surface occurs. Approximately 12 min after the PFV-1 IN injection was stopped, the surface was regenerated by injection of the buffer solution containing 0.1% SDS. As can be seen in Figure 4F to 4H, the PFV-1 IN/DNA complexes were completely dissociated by the regeneration procedure allowing repeated injections of the protein. During the course of the experiment (10 h), no deterioration of the surface was observed.

**Figure 5:**
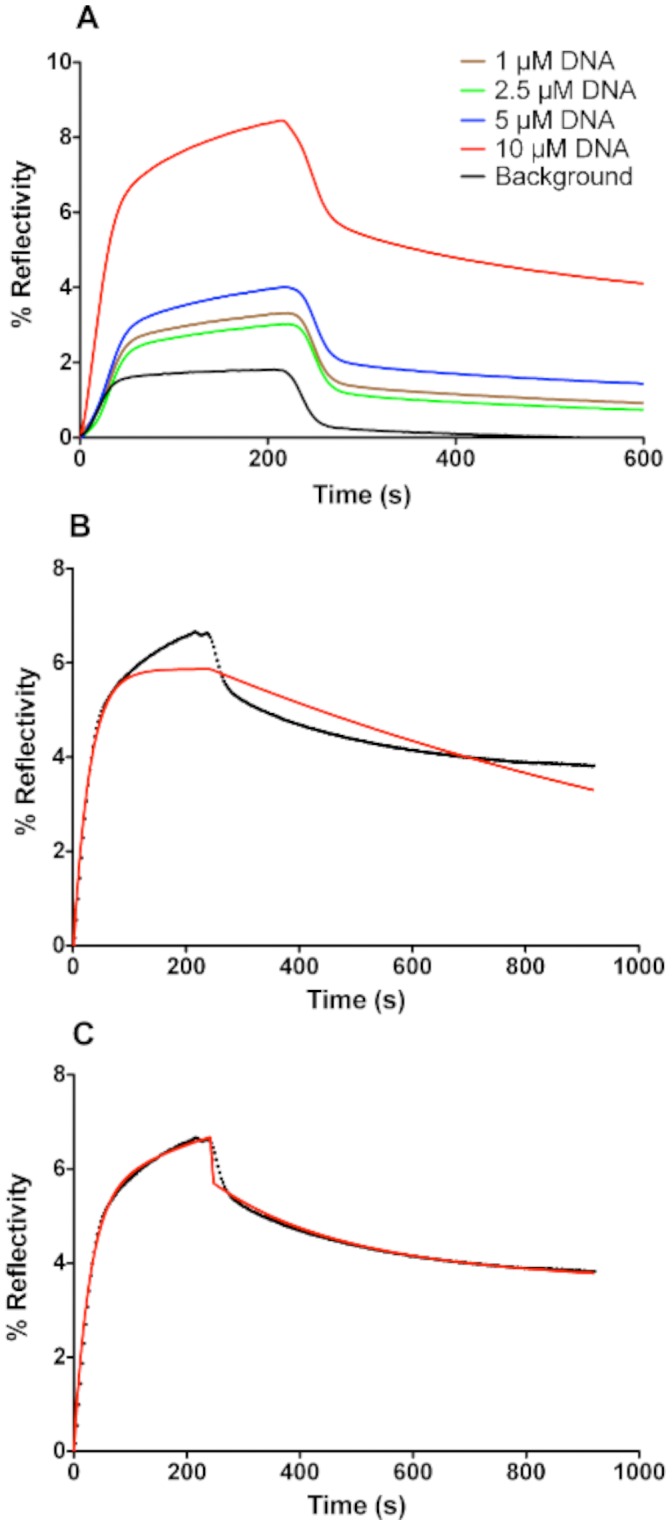
SPRi kinetic curves of PFV-1 IN (200 nM) interacting with immobilized dsDNA on the pre-treated surface. The values for % reflectivity were obtained from direct CCD camera measurements averaged across each spot shown in Figure 5 and as described in [27]. **A**: Changes in % reflectivity at selected spots on the SPRi surface as a function of time as PFV-1 IN passes over the prism surface. The curves show binding to spots containing different concentrations of DNA (1, 2.5, 5 and 10 µM) or to reference spots (containing no DNA) outside of the zone to which DNA was applied (designated as background). **B**: The kinetic curve after subtraction of the background for reaction taking place on a spot where 10 µM DNA solution was deposited. The red line is a fit carried out by applying a single exponential model 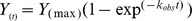 where 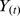 is the % reflectivity at time *t*; 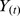 is the amplitude of the phase, and the observed rate constant 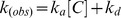, 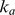 is the association rate constant, 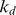 is the dissociation rate constant calculated from a simple exponential fit of the dissociation phase using 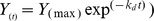 and [*C*] is the concentration of PFV-1 IN (200 nM). C: same curve as shown in **B** but fitted (red line) with a double exponential model for both association and dissociation. The model for association is 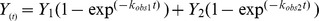 and dissociation is obtained from 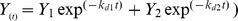 where 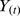
is the % reflectivity at time *t*; and 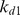 and 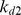 are the respective dissociation rate constants for the two phases 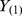 and 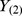; 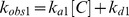 and 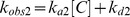.

Kinetic curves, characteristic of interactions between 200 nM of PFV-1 IN and the target DNA, are shown in figure 5A. The maximum intensity of the reflected SPR light depends on the dsDNA density on the surface indicating that the amount of PFV-1 IN captured is, in part, dependent on the DNA density adsorbed on the surface. The reference curve corresponding to the reflectivity measured on the surface around the spots is characterized by a very small increase in reflectivity during the association step and an immediate drop to a very low reflectivity immediately after the protein injection stops, indeed the signal is so weak and transient that calculation of kinetic constants is beyond the sensitivity of the technique used here; consequently this signal is interpreted as being due to a very weak non-specific interaction with the surface presumably as molecules roll across the surface without establishing a specific molecular interaction.

**Figure 6:**
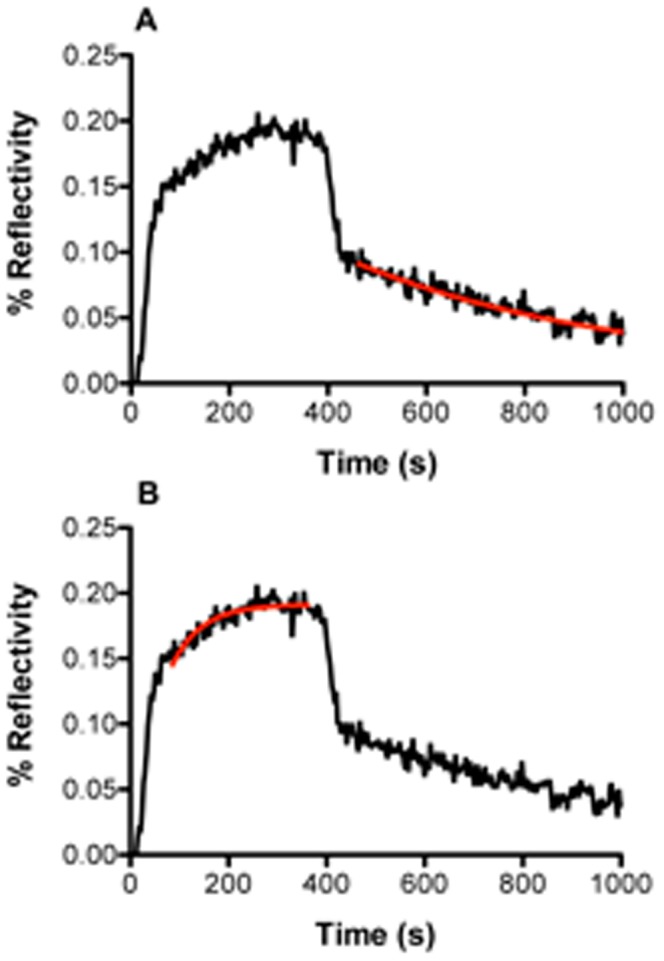
(5 µM) on the pre-treated surface. The curves, (generated as described in the legend to Figure 6) were fit to: **A**: a single exponential dissociation model (red line) or **B**: a single association model (red line). Calculated rate constants from these fits gave the following values: k_a_ = 3.7 10^6^ M^−1^s^−1^; k_d_ = 1.8 10^−3^s^−1^; K_D_ = 4.9 10^−10^ M.

**Figure 7:**
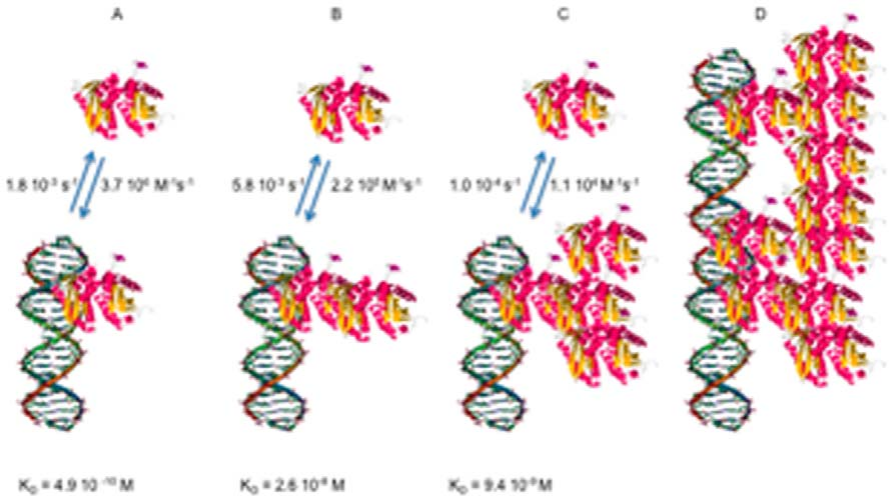
Cartoon describing the model for PFV-1 IN binding to DNA and subsequent accretion of proteins. **A**: PFV-1 IN binds to the DNA; **B**: the nucleoprotein complex then binds more protein; **C**: which in turn continues to oligomerise until, **D**: the protein completely covers the DNA. The molecules are not to scale nor do they depict actual known orientations. The rate and equilibrium constants associated with B and C may be interchanged, as the model does not allow attribution of either to the structural forms suggested. The PFV-1 IN is represented in the monomeric form for simplicity.

Direct fitting of the curves (collected on the spot where 10 µM dsDNA solution was deposited) after subtraction of the reference curve, using a simple Langmuir analysis is shown in Figure 5B; under these conditions the fit was very poor and clearly the interaction is more complex than could be described by a simple one to one binding model. We therefore fit the association and dissociation phases to a two-phase model as described in the legend to figure 5 and extracted constants that are shown in table 1 (Figure 5C). It should be noted that particularly at low PFV-1 IN concentrations a somewhat sigmoidal form of response curve during injection is observed; this is due to the effect of the sensor cell design on the flow across the surface and is generally removed by subtraction of the reference curve (i.e. obtained from a region of the surface where no DNA has been spotted as shown in the difference signals) or by ignoring a short initial part of the injection phase. There was no observable effect of dsDNA density on the values of the kinetic constants (not shown). The most striking feature of this analysis apart from the fact that PFV-1 IN is binding with multiple modes to a random dsDNA sequence is that there is a phase with a fast association rate and fast dissociation rate corresponding to an equilibrium dissociation constant K_D1_ of 2.6×10^−8^ M and a second phase with a slower association rate and slower dissociation rate corresponding to a K_D2_ of 9.1×10^−9^ M. In this work we make no assumption concerning the multimeric form of the protein and hence express all concentrations in terms of monomer of PFV-1 IN. Fundamentally we observe a multiphase process that we limit to an analysis of two phases. Clearly however, since the amount of non-specific binding of the protein to the surface is negligible in our system, it does not contribute to the detected measurements (figures 4 and 5). The resulting kinetic constants are therefore strictly characteristic of the binding of PFV-1 IN to dsDNA and the extracted kinetic rates are exclusively representative of interactions of the PFV-1 IN with the target dsDNA. However, given that we observed a phase with a low nano-molar K_D_ for binding, we decreased to 4 nM the concentration of PFV-1 IN in order to favor this binding mode alone (Figure 6). As expected, at this concentration PFV-1 IN binding to 5 µM dsDNA spots was now essentially monophasic. The background signal was essentially non-existent so a background value was not subtracted which explains the bulk effects seen at the beginning and the end of the injection period. These bulk effects are simply due to the difference in the composition of the reaction buffer due to the dilution of the protein into the reaction buffer giving rise to a slightly different bulk refractive index which is seen as a sharp rise and fall during the onset of the association and dissociation phases respectively. Calculated rate constants, shown in the legend to Figure 6 were different from those reported in Table 1 obtained at higher (200 nM) concentrations of PFV-1 IN and led to an affinity constant of 4.9 10^−9^ M. Of importance also is that the order of magnitude of the binding response (% reflectivity around 0.2) was at least 20 times lower than that observed for 200 nM PFV-1 IN binding to the same surfaces (% reflectivity around 4–5). The binding phenomena observed here therefore, is not the same as that observed at higher PFV-1 IN concentrations. We used a rather simplistic fitting model involving a minimum of two sites although clearly a number of potentially different sites may be involved, applying more sophisticated fitting procedures such as a stretched exponential (Kohlrausch) kinetic approach gave poor fits with admittedly beta functions inferior to 1 but probably unsuitable for the present situation where a number of non overlapping binding sites are available rather than a complex reaction involving a chain of intermediates undergoing a succession of conformational changes. As a consequence staying with a simple two sites binding mode we propose the model outlined in Figure 7. At low concentrations of PFV-1 IN the protein binds to the DNA (bear in mind that the DNA does not contain specific binding sites *per se* for PFV-1 IN). As the concentration of PFV-1 IN increases however we suggest that subsequent binding of the protein is also mediated through protein/protein interactions and through at least two modes, one via an interaction between DNA bound PFV-1 IN and via an interaction with protein that has already bound to DNA-bound PFV-1 IN. In this model we have arbitrarily attributed these latter two phases to one or the other of these steps although there are no data to support this at the moment and the constants associated with steps **B** and **C** (Figure 7) may in fact be interchanged. In the cartoon of Figure 7 the PFV-1 IN is represented in the form of a monomer unit as the monomer-dimer equilibrium for the protein alone has a half-transition concentration of 20–30 µM [18]; were this unit form to be monomer, dimer or tetramer should have no impact on the model. This model therefore would explain the three phases seen in Figures 5 and 6, and furthermore explain why we observe binding modes at lower concentrations of PFV-1 IN that differ considerably from those observed at higher concentrations of PFV-1 IN both in this report and also in the published literature (Apparent K_D_ = 400 nM at 150 mM NaCl) [18]. This model would also imply that studying PFV-1 IN/DNA interactions at high (>10 nM) concentrations using techniques that look simply at mass or bulk increases (SPR, anisotropy, etc.) would miss the initial binding event and simply report subsequent oligomerisation on the DNA. The predicted K_D_ values in the sub nM range also are more consistent with predicted concentrations of the active form of PFV-1 IN in infected cells since it was estimated that there are only about 50 to 100 copies of each protein [25].

**Table 1:** Calculated rate constants from fits of the type shown in Figure 5C.

k_a1_M^−1^s^−1^	k_a2_ M^−1^s^−1^	k_d1_ s^−1^	k_d2_ s^−1^	K_D1_ M	K_D2_ M
2.2±1.4•10^5^	1.1±0.8•10^4^	5.8±0.8•10^−3^	10.0±4.0•10^−5^	2.6•10^−8^	9.1•10^−9^

Values are shown based on binding to the four DNA concentrations spotted on the surface; errors refer to standard error for the rate constants calculated for each DNA concentration. The equilibrium dissociation constants K_D1_ and K_D2_ were calculated from the ratio of the respective kd/ka values.

Thus our observation of at least three binding modes of PFV-1 IN to a random dsDNA sequence for which it should have no target specificity may be explained by an initial binding of the PFV-1 IN moiety to DNA followed by further, slower interactions of the nucleoprotein complex with PFV-1 IN to form larger, more stable complexes [26].

To our knowledge the development of these supports for biosensor surfaces provides access to the study of specificity of PFV-1 IN binding to DNA under conditions that closely mimic the *in vivo* situation in terms of PFV-1 IN expected concentrations.

## Discussion

PEG based antifouling surfaces and similar chemical strategies are the subject of intense research that seeks to circumvent a major problem of non-specific binding to biosensor surfaces. In this paper we show that the antifouling properties of EG*4* based SAMs coupled with the dynamics of SAM adsorption allow control of the target DNA adsorption in terms of stability and density. The originality of the method resides in protecting the gold surface with a SAM adsorbed for very short times such that the resulting monolayer is not well packed and poorly, if at all, organized. We show that despite the presence of a large number of defects the resulting SAM based on EG*4* chemistry is highly efficient in preventing non-specific adsorption of the protein on the surface and allows optimization of the DNA arrays for use on SPRi.

We further demonstrate the use of our surfaces to obtain apparent kinetic rate constants for a difficult system notably that of the formation of nucleoprotein complexes between the PFV-1 IN protein and DNA. These surfaces thus permit surface based characterization for this type of interaction in which non-specific binding to non-target molecule bound surfaces is effectively eliminated, thus obviating the necessity for ambiguous background subtraction. The use of this two step SAM adsorption for the analysis of the formation of nucleoprotein complexes involving proteins that bind with a high degree of non-specificity to non-target supports is clearly of great potential especially with respect to investigating the specificity of binding of PFV-1 IN to its cognate target. This is clearly a complex process and we are currently applying this approach to characterizing those elements responsible for LTR selectivity exhibited by PFV-1 IN.

## Materials and Methods

### DNA Sequence and Adsorption Protocol

43 base thiolated ssDNA oligonucleotides (MWG Eurofins) were kept in their oxidized form DNA-(CH_2_)_6_-S-S-(CH_2_)_6_-DNA in order to protect the thiol group from forming undesired oxidation products. Each oligo contained a mercapto-hexane needed for chemisorption on gold surfaces and five thymines - (T)_5_ as a spacer at the 5′end. The full sequence of the thiolated ssDNA was: 5′-SH-C6-TTTTT-CTA-AGC-TAG-AAG-ATT-ACT-CCA-AGT-ACA-TAA-TCT-AAA-AT-3′. The 38 base complementary strand (5′-AT-TTT-AGA-TTA-TGT-ACT-TGG-AGT-AAT-CTT-CTA-GCT-TAG-3′) was hybridized with the thiolated strand at a molar ratio of 1∶1.1 of thiolated to non-thiolated strand respectively. The resulting dsDNA corresponds to a random sequence that should not represent a specific target for INT binding. Prior to adsorption, the desired amount of DNA was incubated with 10 mM of the reducing agent Tris(2-carboxyethyl) phosphine (TCEP) in 0.4 M NaH_2_PO_4_, pH 7.4. The mixture was incubated at room temperature for several hours to allow complete reduction of the disulphide bonds. The DNA samples were then passed through a column (BioSpin 6, BioRad) pre-equilibrated with the buffer used for the DNA adsorption (0.4 M NaH_2_PO_4_, pH 7.4). The high molecular weight DNA molecules were collected in the flow-through in adsorption buffer. The final dsDNA solution was adjusted to the desired concentration. The flow-through samples were spotted immediately onto freshly pre-treated gold surfaces.

### Preparation of DNA Chips

DNA chips were prepared on a glass prism (SF 10 with a high refractive index: *n* 1.707987 at λ = 830 nm) activated by Reactive Ion Etching (RIE) prior to the thermal evaporation of a 50 nm gold layer. First, a fresh gold layer was immersed in a solution containing 0.1 mM of 1-undecanethiol substituted with a hydroxyl-terminated tetra(ethylene glycol) (EG*4*-OH) diluted in ethanol for times ranging from 30 s to 2 hrs at room temperature. Before DNA deposition the prisms were thoroughly rinsed in pure ethanol for 20 min. The DNA solution was spotted (using a Hamilton robot) on the freshly pre-treated prism surface at different concentrations as indicated in the text (from 0.1 µM to 10 µM in phosphate buffer (0.4 M) at pH 7.4) and incubated for 16 hrs in a sealed Petri dish at 100% relative humidity to prevent the DNA solution from drying. Moreover, in order to reduce evaporation during incubation, the DNA solution contained 5% glycerol. The prism was then directly inserted into the SPRi apparatus and the buffer selected for PFV-1 N/DNA binding assays (PFV-1 IN buffer: 20 mM Hepes pH 8, 10 mM MgCl_2_, 100 mM NaCl) was immediately flowed across the surface at 25 µl/min. To remove any contaminants from the surface, 200 µl of SDS diluted to 0.1% (w/v) was injected across the surface at 25 µl/min. Small pinpoints within each spot seen in the deferential images (see figure 1 and 4) are due to contact made by the pintool of the robotized spotting process that physically locally damaged the thin 50 nm gold surface.

### DNA Radiolabeling

The thiolated DNA strand was labelled at its 3′ end using the terminal deoxytransferase TdT (New England Biolabs) and 3′-deoxyadenosine 5`-triphosphate-[α-^32^P] (Perkin Elmer) and the non-thiolated DNA oligomer was labelled at its 5′ end using the enzyme T4 polynucleotide kinase (New England Biolabs) and [^32^P]-*γ*-ATP (Perkin Elmer) according to published protocols. The radiolabeled ssDNA strands were hybridized with their complementary non-labelled strands at a ratio of 1∶1.1 of thiolated to non-thiolated strand, respectively, to ensure that all of the thiolated strands were hybridized. The oligomers were incubated at 80°C for 10 min, following slow cooling to room temperature. The efficiency of hybridization was evaluated by loading samples of the radiolabeled single stranded DNA and double stranded DNA on a non-denaturing 15% polyacrylamide gel, at a 1∶19 ratio of bisacrylamide to acrylamide in 89 mM Tris borate, 2 mM EDTA (1×TBE buffer). Under these conditions the migration of the double strands is retarded compared to that of the single strands.

### Quantification of DNA Adsorbed onto Pre-treated Gold Surfaces

A solution of 5 µl of [^32^P] DNA at different concentrations (indicated in the text) was deposited on an EG4-OH pre-treated gold surface for 16 hrs. The samples were then rinsed with PFV-1 IN buffer for 10 min and PFV-1 IN buffer (20 mM Hepes pH 8, 10 mM MgCl_2_, 100 mM NaCl) containing 0.1% SDS for an additional 10 min before letting the samples dry to air. The specific activity of each DNA sample is defined as the amount of radiation produced by a known quantity of that DNA. This number was determined by spotting 1 µl samples of radioactive DNA onto a gold reference slide allowing them to air dry without any washing, followed by quantification by phosphor-imagery (Typhoon Trio, GE Healthcare). The amount of pixels at each spot was divided by the amount of pmoles pipetted, yielding the specific activity of the samples.

### SPR Imaging Setup

The biological interface consists of a prism surface coated with a thin layer (∼50 nm) of gold. An evanescent field called a plasmon wave is created at the interface of this gold-coated surface and the dielectric from a light beam arriving through the prism at an angle of total internal reflection (TIR). At TIR there is a resonance effect measured by imaging the entire reflected light from a monochromatic polarized electroluminescent diode using a camera linked via a dedicated optical system. Consequently this allows analysis of an entire surface upon which discrete spots of ligand are immobilized. A microcuvette system allows material to be flowed across the surface and the SPR response at predetermined spots can be assessed in parallel by a time resolved CCD that captures changes in percentage reflectivity at selected spots on the surface. Changes in percentage reflectivity averaged across the surface of each spot as a function of time can be related to changes in concentration of mass at each spot, thus giving access to the kinetics of interactions at the surface at each spot. Most importantly, non-specific interactions of the molecules directly with the surface around selected spots can be simultaneously quantified and compared with specific interactions occurring with the target material in the spots, assuming that the amount of non specific interaction inside and outside the spots are identical. A recent work has shown that kinetic constants extracted from the kinetic curves collected with a Biacore SPR apparatus or from the SPRi apparatus are comparable when using identical surfaces and conditions [7].

SPRi data were collected using a SPRi apparatus purchased from GenOptics (Orsay, France) with an incoherent light source (*λ* = 830 nm) as described previously [7]. This optical method is sensitive to small changes in the refractive index near the gold layer (exponentially decreasing to a penetration depth of ∼200 nm). SPR images captured by a time resolved CCD camera and LabView software (GenOptics, France) permitted real-time averaging of the intensity on each spot in order to obtain the reflectivity signals.
